# Supplementary material for: Refined weathering CO2 budget of the Tibetan Plateau strongly modulated by sulphide oxidation
Source: Nat Commun. 2025 Mar 20;16:2741. doi: 10.1038/s41467-025-58046-4 (PMC11926071; doi:10.1038/s41467-025-58046-4)
Supplement: Supplementary file 1 — Supplementary Information [file 41467_2025_58046_MOESM1_ESM.pdf]

Supporting Information

Refined weathering CO_2 budget of the Tibetan Plateau strongly modulated by sulphide oxidation

Wenjing Liu[a,b], Zhifang Xu[a,b*], Huiguo Sun[a,b], Mingyu Zhao[a,b], Yifu Xu[a, b], Zhengtang Guo[a,b]

[a] State Key Laboratory of Lithospheric and Environmental Coevolution, Institute of Geology and Geophysics, Chinese Academy of Sciences, Beijing 100029, China

[b] College of Earth and Planetary Sciences, University of Chinese Academy of Sciences, Beijing 100049, China

*Corresponding author. Tel./fax: +86 10 82998289

E-mail address: zfxu@mail.iggcas.ac.cn (Zhifang Xu)

Supporting Text A: The Description of Studied Large River Basins Developed on the Tibetan Plateau

The Tibetan Plateau extends approximately 2,500 kilometers (km) from east to west and 1,000 km from north to south, with an area of ~2.5×10^6 square kilometers (km^2), which accounts for about two percent of the earth land surface[1]. The plateau average elevation exceeds 4,000 meters a.s.l., and has the largest glacial ice volume and permafrost area outside the polar regions[2-3]. Furthermore, all the large river systems (e.g. the Ganges-Brahmaputra, the Indus, the Yellow River, the Yangtze River, etc.) in Asian continent are originated from here. The plateau is bordered with the Kunlun Mountains and its associated ranges to the north and the Himalayas and Karakoram Range to the south and southwest.

Formed by a continental collision between the Indo-Australian Plate and the Eurasian Plate, the lithology background is complicated and heterogeneous. Generally, bedrock on the Tibetan Plateau consists mainly of paleozoic carbonate and clastic rocks; with volcanic rocks scattered in the suture belts at the edge of the plateau[4-6]. In the northern part of the Tibetan Plateau, there is mainly deposited late Cenozoic sedimentary rocks and a range of faulted Triassic shale and sandstone along the Kunlun Mountain[1]. While in the southern Tibetan Plateau, bedrocks are mainly composed of carbonate and igneous granite gneiss, with evaporites can be found in the relatively dry area of the plateau, usually in the upper reaches of the river systems[4]. With continuous Indus Tsangpo Suture tectonic movements near the Eastern Syntaxis, calc-alkaline plutons in this zone are highly metamorphosed and surrounded by quartzites, phyllites and marbles[6].

The Tibetan Plateau has a complex climate system and strong environmental gradient. Overall, it is characterized by cool-dry winters and wet-humid summers[7]. Approximately 60~90% of the annual total precipitation happened from June through September leading by the Indian monsoon season. The annual precipitation increases from less than 100 mm in the north-west plateau to above 800 mm in the south-east plateau[8]. The annual average temperature is lower than 0 °C on the northern plateau and ranged from 0 °C to 5°C in the western part. While in the Yarlung Tsangpo River basin of the southern Tibetan Plateau, annual air temperature is higher than 5 °C.

In this work, about 200 river water samples were collected from the mainstream and major tributaries of all the large rivers basins developed on the Tibetan Plateau (Fig. 1, Table S1). The studied rivers could be classified into two systems, the Indian Ocean River System (IOWS) and the Pacific Ocean River System (POWS). The former includes Nu Jiang (upper reaches of Salween River) and Yarlung

Tsangpo River (upper reaches of Brahmaputra River), and the latter includes the upper Yellow River, the upper Yangtze River and Lantsang Jiang (upper reaches of Mekong) River. The upper reaches of the Yangtze River draining on the plateau were usually divided into several parts/tributaries, namely Tongtian River, Jinsha river, Yalong River and Dadu River.

Supporting Text B: Solute Source Identification and Contribution Flux Calculation for Major Rivers in the Tibetan Plateau

Ⅰ. The forward model description and calculated results for the river solute contributions from different sources.

A forward model was adopted to quantify the river solute contributions from different sources. The dissolved solids in river water of the Tibetan Plateau could be ascribed to the solute products of atmospheric precipitation, anthropogenic input, carbonate and silicate rock weathering, evaporite dissolution and sulfide mineral weathering within each drainage basin. Thus, for any element X in the river water, its budget equation could be written as

$$[X]_{river} = [X]_{atm} + [X]_{anth} + [X]_{carb} + [X]_{sil} + [X]_{eva} + [X]_{sulf} \quad (1)$$

where subscripts “atm”, “anth”, “carb”, “sil”, “eva”, and “sulf” represent inputs from the six contributors on the plateau above.

Cl^- is the most commonly used reference when evaluating atmospheric inputs to river water. In the pristine areas, the concentration of Cl^- in river water is normally assumed to be entirely derived from the atmosphere, assuming that the contribution of evaporites is negligible[11-14]. Furthermore, the proportion of atmosphere-derived ions in river water could be calculated by the X/Cl^- ratios of the rainwater. However, the large variations of rainwater chemistry have been an important source of error in estimation of the atmospheric contribution to the riverine solutes and atmospheric chemistry data is quite limited in the plateau. We compile all the rainwater chemistry data available in and around the plateau and calculate the mean Cl-normalized molar ratios for different basins on the plateau (Table S2)[15-25]. Corrections for atmospheric inputs are able to be made for each of the studied basins by assuming that the sample with the lowest Cl^- concentration in each basin has gotten its entire Cl^- from rainwater. Evaporite (halite and gypsum) dissolution has been documented to be an important contributor of dissolved loads in the rivers draining on the Tibetan Plateau, especially for the headwaters[10,26-28]. The solute contributions from evaporite (chloride and sulfate salts) are calculated after atmospheric input correction by assuming that the remaining Cl^- in river water after atmospheric input deduction are from halite ($Cl_{evap} = Cl_{riv} - Cl_{atm}$)

and these Cl^- are balanced by Na^+. In addition, the riverine SO_4^{2-} after atmospheric input deduction are assumed deriving from gypsum dissolution and the oxidative weathering of pyrite (OWP). The Ca^{2+}/Na^+ ratio of 0.35±0.15 and Mg^{2+}/Na^+ ratio of 0.24±0.12 for the silicate weathering end-member by ref. 24 are adopted to calculate the contribution of Ca^{2+} and Mg^{2+} from silicate weathering, and the residual Ca^{2+} and Mg^{2+} are attributed to be from carbonate weathering. Based on the lowest Cl- concentration and the X/Cl^- ratios of the rainwater in each basin, it is calculated that 0.1~19.6% of the total cations in the different plateau rivers are derived from atmospheric precipitation (Supplementary Text A and Table S3). The majority of water samples (189 out of 200) are with atmospheric contribution lower than 5%.

The Qinghai-Tibet Plateau has harsh living condition and is sparsely populated. As a typical indicator of anthropogenic activities, the average riverine nitrate concentrations are as low as 22.0 μmol L^{-1} (Table S1), much lower than the value of river waters impacted by human disturbance, e.g., 159 μmol L^{-1} for lower reaches of the Yellow River[29]; 104 μmol L^{-1} for the Mississippi River[30]; 185 μmol L^{-1} for the Elbe River[31]. Therefore, it is reasonable to consider a relative minor and negligible contribution of anthropogenic sourced materials to the major ion budgets compared with other contributor for the studied rivers, which has also been well documented in previous surface water studies in the plateau[32-34].

After the calculation of atmospheric inputs, the correlations between HCO_3^-/Na^+ and Mg^{2+}/Na^+ vs. Ca^{2+}/Na^+, and $^{87}Sr/^{86}Sr$ ratios vs. Sr^{2+}/Na^+ are applied to identify the solute origination of the plateau rivers (Fig. S3). The large river water samples in this study fall into the space constructed with evaporite, carbonate and silicate weathering end-members defined by the 61 world large rivers (ref. 35) in all the plots in Fig. S3, which indicates that the solutes of these rivers are the mixture of the dissolved products from these rock types. However, the higher Mg^{2+}/Na^+, Ca^{2+}/Na^+ and HCO_3^-/Na^+ ratios for the rivers in the plateau compared with the global large rivers suggests a stronger influence of carbonate weathering inputs on river water chemistry.

The solute contributions from evaporites (chloride and sulfate salts) are calculated after atmospheric input correction, and the silicate and carbonate weathering contributions to the riverine total cationic loads in the Tibetan Plateau are calculated with the end-member appointments and deducing procedures above. The calculated contributions (in %) from the different reservoirs in the plateau to the total cationic loads are presented in Supplementary Table S3 and Fig. 2. The Jinsha River in the northeast of the plateau has significantly higher solute contribution from evaporite dissolution (averaging at 59.7% for the mainstream water) compared with other rivers. While the Yalong and Dadu river located at the eastern

edge of the plateau have the lowest evaporite contributions in the range of 0~20.4% (with most samples lower than 5%), and the highest carbonate weathering contribution ranging from 16.1% to 91.5%.

Ⅱ. Source Identification of Riverine DIC

Riverine DIC content and carbon isotopic signals carry the integrated geochemical characteristics of HCO_3^- produced by different weathering interactions in a basin, i.e. carbonate and silicate weathering by carbonic and sulfuric acid. The theoretical $\delta^{13}C$ values of DIC derived from different weathering interaction pathway accomplished by different acid agents and rock types has been well documented in previous research. Soil CO_2 serves as a key link in the DIC generation and transportation system, as it is the major acid agent involved in carbonate and silicate weathering[36-39]. Based on the theoretical and previous reported endmember value, the $\delta^{13}C_{DIC}$ and its co-variation with SO_4^{2-}/HCO_3^-, $HCO_3^-/Ca^{2+}+Mg^{2+}$, and Si/HCO_3^- ratio are employed to illustrate the involvement of carbonic and sulfuric acid and the role of silicate and carbonate weathering in contributing to DIC of the large river systems in Tibetan Plateau (Fig. 4). The water chemistry and $\delta^{13}C_{DIC}$ from the studied mainstreams and main tributaries fall within the area defined by the boundaries of silicate weathering by carbonic acid and carbonate weathering by carbonic and sulfuric acid, with a majority of samples lying in the mixing area of carbonate weathering by both acid in all the plots in Fig. 4. The ref. 93 reported that the $\delta^{13}C_{DIC}$ value in rivers on the Qinghai Tibet Plateau is significantly higher than that in the densely vegetated area, indicating a potential significant contribution of carbonate weathering to DIC in the catchment and the limited contribution of soil respiration under dry climate and sparse vegetation. Therefore, it is obligated to fully and quantitatively illustrate the weathering pathway, and the acid agent sources in the chemical weathering of the plateau, before we can get an accurate estimation on its atmospheric CO_2 source and sink effects.

Ⅲ. Quantification of Riverine Sulfate Source Contribution

Partitioning the riverine sulfate sources is the premise to estimate the portion of protons originating from sulfides oxidation and to discriminate cations released by carbonic and sulfuric acid weathering, thus the corresponding CO_2 consumption fluxes[4,26,32,36,40-41]. The main sources of sulfate in river are commonly composed with atmospheric deposition, gypsum dissolution, sulfide mineral oxidation and anthropogenic input. The $\delta^{34}S_{SO4}$ is plotted with SO_4 concentrationfor river waters in the large rivers in the Tibetan Plateau in Fig. S4[27-28,42-50]. The $\delta^{34}S_{SO4}$–$1/SO_4$ scatter diagram (Fig. S4) indicate that the riverine SO_4^{2-} are the mixture of atmospheric deposition, gypsum dissolution, and sulfide mineral

oxidation products. The average $\delta^{34}S_{SO4}$ values (2.6‰) of the large rivers in the Tibetan Plateau in this study are lower than the estimation of the global riverine $\delta^{34}S_{SO4}$ (4.4‰, ref. 51), implying a more significant contribution from endmembers with a light sulfur isotopic composition compared with other large rivers worldwide, most possible from pyrite weathering considering intensive exposure of the sulfide mineral due to the high erosion rates in the plateau.

$\delta^{34}S_{SO4}$ and $\delta^{18}O_{SO4}$ could be employed to discern the sulfate sources in river systems based on the distinct isotopic compositions in different sources and to calculate the portion of riverine sulfate sourcing from dissolution of sulfate minerals in evaporites versus oxidative weathering of pyrite (OWP)[27,39,44-45,49,52]. The proportion of the sulfate derived from rainwater, gypsum dissolution and oxidation of pyrite could be calculated as follows:

$$SO_{4river} = SO_{4rain} + SO_{4gypsum} + SO_{4pyrite} \quad (2)$$

$$\delta^{34}S_{river} = f_{rain} \times \delta^{34}S_{rain} + f_{gypsum} \times \delta^{34}S_{gypsum} + f_{pyrite} \times \delta^{34}S_{pyrite} \quad (3)$$

$$1 = f_{rain} + f_{gypsum} + f_{pyrite} \quad (4)$$

where f is the proportion of the sulfate derived from rainwater, gypsum dissolution and oxidation of pyrite. The riverine SO_4^{2-} after atmospheric input deduction (calculated in Supporting Text B Ⅰ) are assumed deriving from gypsum dissolution and OWP. Therefore, it is critical to accurately define the $\delta^{34}S_{SO4}$ value of these end-members for the investigated area before calculating their contribution to the riverine SO_4^{2-} and explore the role OWP playing. For the atmospheric input, it has been reported that the average rainwater/particle $\delta^{34}S$ values in China mostly fall into the range of 0 ~ 5‰, and the $\delta^{34}S$ value of atmospheric particles at Waliguan station in the Tibetan Plateau and in the Sichuan Basin at the east of the Tibetan Plateau are averaged at 4.2‰ and 3.9‰, respectively[53-54]. The $\delta^{34}S$ and $\delta^{18}O$ value of gypsum varies in a wide range from 10‰ to 30‰ and from 10‰ to 20‰, respectively[37,55-56]. In this study, the $\delta^{34}S_{SO4}$ and $\delta^{18}O_{SO4}$ isotopic values of stream water in the upper reaches of Huang, Jinsha, Yalong and Nu River basin where evaporites widely distribute are in a relatively narrow range of 8.31 to 10.8‰ and 9.81 to 15.44‰, respectively (Supplementary Table S1). These ranges are in consistent with previous investigation of evaporite $\delta^{34}S_{SO4}$ in Yalong river (16‰ and 12‰, ref. 37) and the Yellow River (16.4‰ and 10.9‰, ref. 26). The $\delta^{34}S$ compositions value of sulfide minerals have been documented to be highly variable, e.g. igneous and sedimentary sulfide minerals commonly have $\delta^{34}S$ values between 10 and -10‰ and between 10 and -50‰, respectively[37,57]. Triassic sediment widely distributed in the northeastern plateau has been regarded as a major sulfide mineral sources, and the $\delta^{34}S$ values in pyrite

from the Triassic stratum are reported in the range of −42‰ to −3‰[58]. In the Zaduo country of Yushu (the center of the studied area, Fig. 1), the $\delta^{34}S$ values of the sulfide mine range from -29‰ to 6‰ with peak range of -8‰ to -6‰[27,59]. The average value of pyrite $\delta^{34}S$ has been reported at ~-4.95‰ in the sedimentary rocks of the Yamdrok-Tso basin in the south of the Tibetan Plateau[50]. The $\delta^{34}S_{SO4}$ sourcing from the oxidation of sulfides is reported at ~-14‰ in the Yalong River[38] and ~ -10 to -5‰ in Huang River basin[50]. Based on the $\delta^{34}S_{SO4}$ value for potential endmembers discussed above, the $\delta^{34}S_{SO4}$ for rainwater, gypsum dissolution and pyrite oxidation are assigned to be 4±2‰, 17.5±1.5‰ and -8±3‰ in the Tibetan Plateau in this study.

The calculation results show that the SO_4^{2-} in the river water of the large rivers in the Tibetan Plateau mainly derives from the oxidation of sulfide minerals and the dissolution of gypsum, while the contribution of rainwater is generally lower than 10% (Table S3). The headwaters of the Huang, Jinsha and Nu River, locating in a dry climate and with widely evaporites distribution, have the highest percentage of gypsum dissolution contribution (with the calculated f_{gypsum} over 60%). The Yarlung Tsangpo River draining the southern part of the Tibetan plateau tops the f_{pyrite} at ~79% for the main channel, and the f_{pyrite} for other major rivers in the plateau ranges from 25 to 77%. The high erosion rates resulted by the intensive glacier activities within the Yarlung Tsangpo River basin are the primary factors facilitating sulfide exposure and oxidation, which lead to the highest f_{pyrite} among the studied river systems.

Supplementary Figures (Fig. S1-S4)

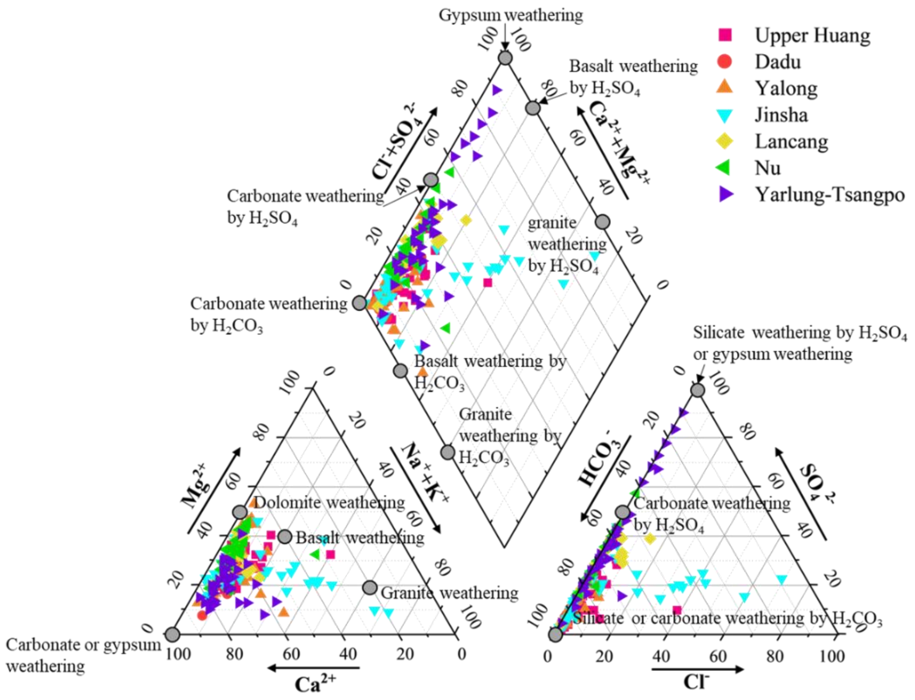

Fig. S1 Piper diagram showing anion and cation compositions of the large rivers in the Tibetan Plateau. The end-member of rock weathering by carbonic acid and sulfuric acid is referenced to ref. 36.

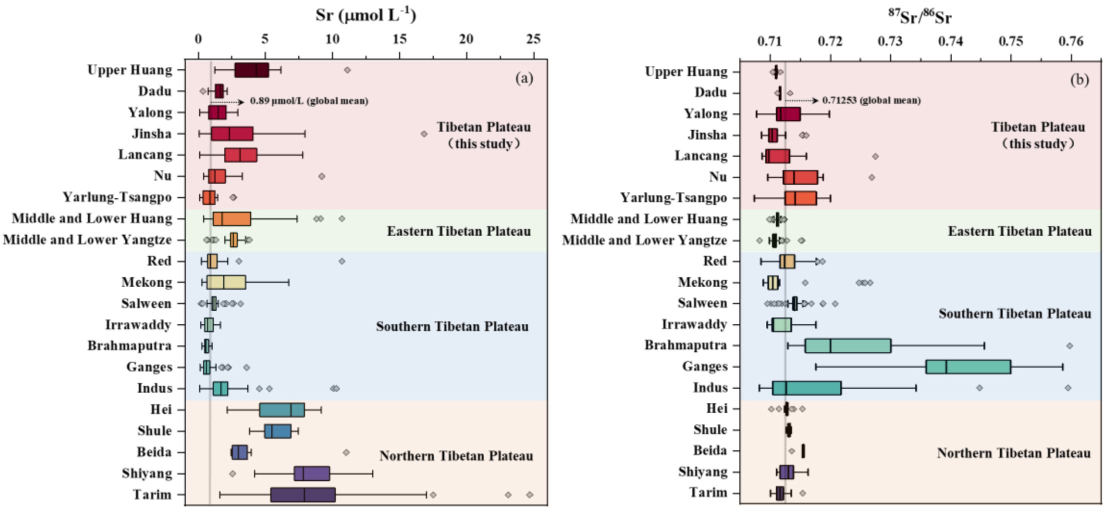

Fig. S2 Box plots of Sr concentration and $^{87}Sr/^{86}Sr$ in rivers on and around the Tibetan Plateau. The datasets for rivers draining on the plateau are investigated in this study. Other data are referenced to refs. 60 and 61-62 for the middle and lower Huang River; refs. 63-64 for the middle and lower Yangtze River; ref. 65 for the Red River; ref. 26 for the Mekong River and Salween River; ref. 66 for the Salween River and Irrawaddy River; refs. 67 and 38 for the Brahmaputra River; refs. 68-69 for Ganges River; refs. 70 and 37 for Indus River; ref. 71 for the Hei River, Shule River, Beida River, and Shiyang River; ref. 72 for the Tarim River; ref. 73 and 35 for the global mean values of Sr concentration and isotope composition.

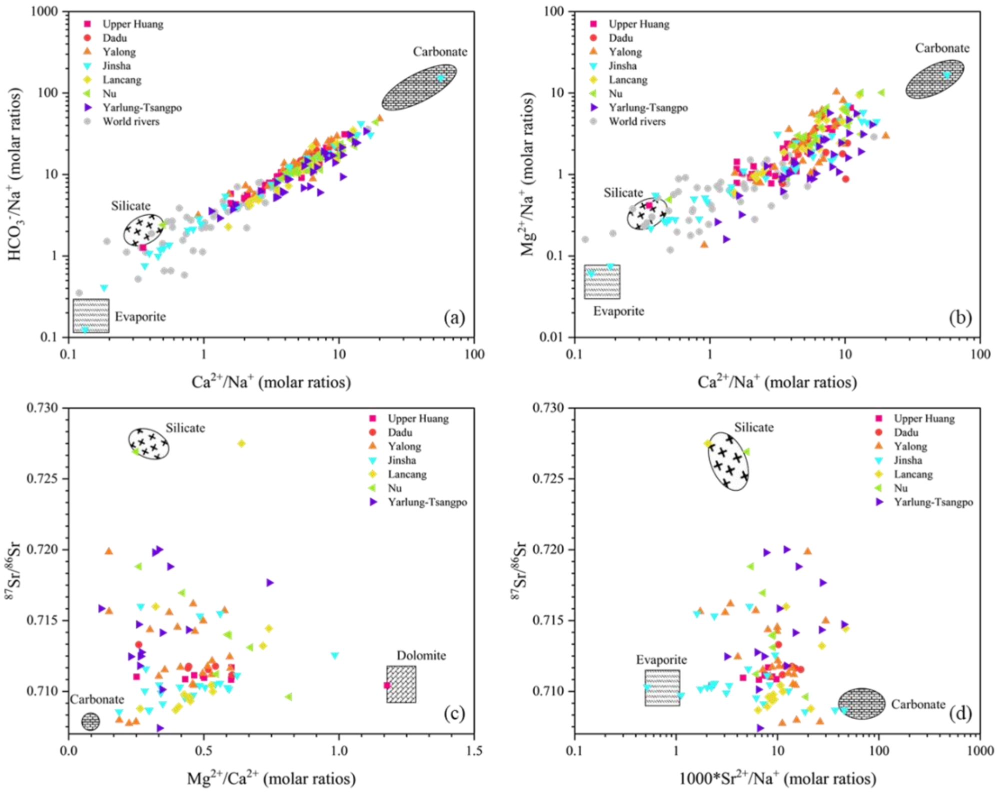

Fig. S3 Correlations between Ca/Na and HCO_3/Na (a) and Mg/Na (b), and between $^{87}Sr/^{86}Sr$ ratios and Mg/Ca (c), and Sr/Na (d) molar ratios of the large rivers in the Tibetan Plateau. The end-member of rock weathering and the world's major rivers data is referenced to ref. 35.

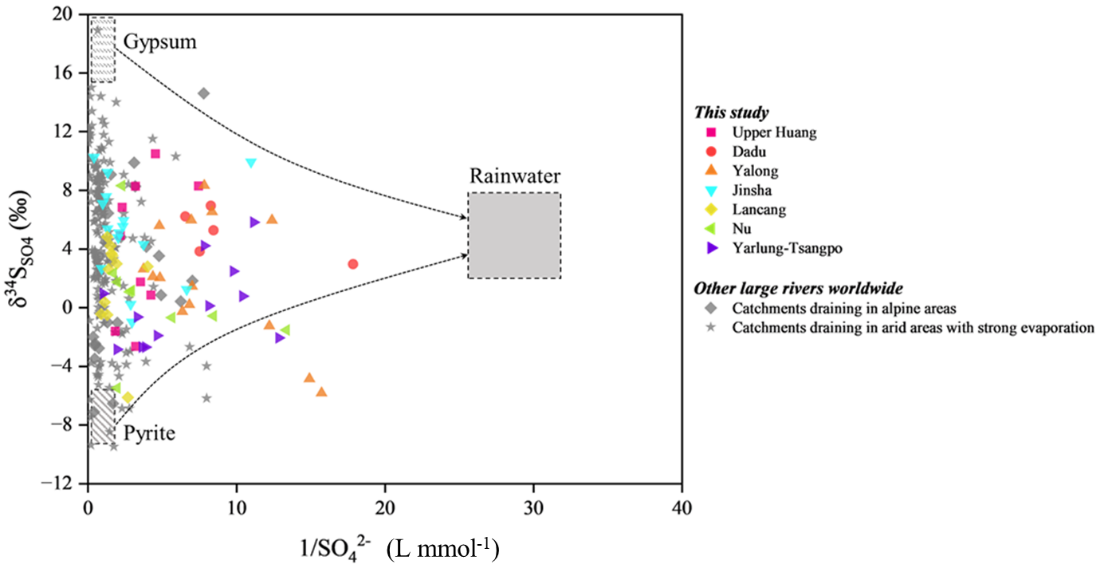

Fig. S4 Correlations between sulfate sulfur isotopes and sulfate concentrations of the large rivers in the Tibetan Plateau and other large rivers worldwide. The sulfate and isotopic compositions of the sources in the diagram were modified after references of refs. 42-44. The data of catchments draining in arid areas with strong evaporation are from refs. 27 and 45-49; The data of catchments draining in alpine area are from refs. 44 and 49-50.

Supplementary Tables (Table S1-S3)

Table S1. Hydro-geochemistry and Sr, C, S and O isotopic compositions of water samples in the studied river networks. Water chemistry data of Jinsha River is from ref. 9; water chemistry and Sr isotope data of Yalong River is from ref. 10.

River	Sample number	T ℃	EC μScm^{-1}	pH	HCO_3^- $\mu mol\ L^{-1}$	F^- $\mu mol\ L^{-1}$	Cl^- $\mu mol\ L^{-1}$	NO_3^- $\mu mol\ L^{-1}$	SO_4^{2-} $\mu mol\ L^{-1}$	K^+ $\mu mol\ L^{-1}$	Na^+ $\mu mol\ L^{-1}$	Ca^{2+} $\mu mol\ L^{-1}$	Mg^{2+} $\mu mol\ L^{-1}$	SiO_2 $\mu mol\ L^{-1}$	TZ+ μEq	TZ- μEq	NICB %	TDS $mg\ L^{-1}$	SPM $mg\ mL^{-1}$	Sr^{2+} $\mu mol\ L^{-1}$	$^{87}Sr/^{86}Sr$	$^{13}C_{DIC}$ ‰PDB	$\delta^{34}S_{SO4}$ CDT ‰	$\delta^{18}O_{SO4}$ ‰VSMOW
Upper Huang	HH-01	13.4	465	8.18	3313	12.92	2436.6	9.8	313	74.1	2606	925	1089	23.7	6709	6399	4.62	447.20	0.32	5.81	0.710433	-3.34	8.27	6.32
	HH-06	16.5	348	8.22	3029	7.11	342.3	92.1	433	35.3	541	1137	683	77.3	4218	4336	-2.81	324.97	0.43	3.55	0.710836	-7.52	6.83	-3.71
	HH-14	17.0	213	7.63	2106	7.63	37.9	21.6	220	32.5	202	881	221	173.1	2438	2613	-7.18	209.40	0.26	1.74	0.711038	-9.9	10.48	10.06
	HH-15	16.9	295	8.17	2698	5.68	178.8	27.9	283	32.7	362	1051	453	90.9	3403	3476	-2.15	268.08	0.15	3.53	0.710866	-11.8	1.74	-1.78
	HH-23	13.4	407	8.43	3216	7.46	387.7	46.9	311	43.3	644	1314	652	94.5	4620	4281	7.33	333.57	0.30	5.00	0.711107	-6.26	-2.67	1.80
	HH-26	17.8	411	8.37	3619	8.87	160.2	42.6	251	45.8	381	1356	739	132.9	4617	4333	6.15	344.20		5.43				
tributary	HH-02	13.0	403	8.30	3491	7.42	545.5	2.7	134	36.5	792	1249	626	70.9	4577	4315	5.73	334.63	0.21	3.61	0.710967	-6.74	8.29	2.15
	HH-03	17.6	463	8.32	3053	4.89	311.1	26.0	737	25.4	335	1767	639	73.7	5173	4869	5.88	369.18		4.14				
	HH-04	16.7	584	8.08	5278	7.34	197.2	45.2	315	62.4	910	1439	1304	85.2	6457	6157	4.66	479.89		5.37				
	HH-05	14.5	278	8.08	2390	4.83	30.2	36.7	194	22.7	212	995	485	84.5	3194	2850	10.77	230.35		2.73				
	HH-07	18.1	502	8.31	4213	5.92	108.9	69.4	399	43.6	395	1471	943	101.6	5267	5194	1.38	402.30		4.61				
	HH-08	17.3	417	8.20	3846	6.27	109.7	40.6	544	42.6	407	1413	851	80.2	4977	5089	-2.25	386.45	0.15	3.29	0.711693	-5.25	-1.63	-2.25
	HH-09	10.6	114	7.77	1124	5.16	27.8	15.3	23	19.1	142	431	136	134.5	1294	1219	5.76	105.50		1.22				
	HH-10	16.7	211	8.05	2095	6.15	29.1	8.8	21	26.8	203	821	229	153.8	2330	2181	6.40	184.91		1.95				
	HH-11	18.9	220	7.79	1231	4.85	36.8	8.8	26	25.2	168	477	131	151.5	1409	1334	5.31	115.77		1.37				
	HH-12	8.9	270	8.37	2689	3.48	9.0	15.8	91	10.5	86	950	570	93.6	3137	2898	7.62	233.99		1.47				
	HH-13	20.5	141	7.69	1278	5.85	27.9	13.0	19	24.4	155	523	135	143.8	1497	1363	8.94	119.10		1.31				
	HH-16	18.2	437	8.27	3574	5.33	64.6	47.6	574	31.8	217	1688	805	90.9	5234	4839	7.55	377.30		4.45				
	HH-17	16.1	537	8.22	3704	4.36	65.5	49.3	1209	33.6	280	2004	1022	82.3	6366	6241	1.96	465.29		4.51				
	HH-18	12.8	425	8.30	4083	9.86	235.7	133.3	159	56.1	419	1756	625	115.2	5236	4779	8.74	385.37		5.18				
	HH-19	15.3	471	8.27	3919	12.36	430.3	138.8	236	56.1	764	1518	705	136.0	5266	4972	5.58	391.70	0.67	5.71	0.711143	-8.68	0.84	6.10
	HH-20	11.1	482	8.32	3874	16.08	316.0	123.7	230	49.3	610	1653	629	116.7	5223	4789	8.31	382.04		5.22				
	HH-21	12.9	532	8.68	4423	10.02	164.1	46.3	740	69.6	991	1561	1162	101.6	6508	6124	5.89	472.20		11.10				
	HH-22	15.5	397	8.61	2996	11.20	241.1	75.6	459	59.5	696	1199	722	90.9	4596	4242	7.70	329.66	0.72	6.17	0.711047	-10.9	4.90	5.80
	HH-24	7.5	483	8.23	3881	7.69	62.8	29.4	68	27.2	182	1461	643	123.7	4418	4117	6.79	334.35		4.64				
	HH-25	17.1	321	8.11	2958	7.80	143.1	21.2	175	31.0	326	1173	526	125.2	3755	3481	7.30	279.83		3.30				
Dadu	DDH-11	16.9	183	8.10	1574	3.94	10.7	13.9	29	28.3	73	703	131	121.4	1770	1661	6.12	141.58		0.79				
	DDH-12	14.8	239	8.40	2151	3.43	16.3	30.2	121	22.3	142	788	428	120.6	2595	2444	5.82	198.72	0.39	1.70	0.711769	-10.1		
	DDH-18		202	7.53	1819	4.78	17.2	6.3	118	27.0	123	643	284	99.5	2003	2084	-4.04	165.94	0.22	1.84	0.711639	-11.3	5.26	
	DDH-19		189	7.66	1695	5.95	17.8	6.7	133	47.3	112	608	270	98.1	1915	1991	-4.00	158.54	0.93	1.56	0.711762	-12.1	3.83	8.07
tributary	DDH-01	13.7	116	7.40	1101	5.57	26.9	11.8	19	16.6	135	464	104	129.9	1288	1182	8.21	103.37		1.34				
	DDH-02	15.6	171	7.94	1704	11.61	24.4	29.4	28	18.2	126	685	226	121.4	1966	1825	7.19	153.34		1.79				

	DDH-03	15.2	222	8.39	1763	4.52	17.1	100.6	121	12.8	127	672	347	108.3	2178	2127	2.33	171.37	0.19	2.16	0.711546	-9.54	6.94	-8.29
	DDH-04	14.6	194	8.11	1543	6.15	19.3	33.5	63	20.8	133	600	234	125.2	1821	1728	5.14	144.16		1.87				
	DDH-05	14.2	348	8.20	2947	5.23	17.9	35.5	125	25.3	169	1031	627	124.5	3510	3255	7.28	263.56		1.91				
	DDH-06	14.7	276	8.31	2745	4.07	12.7	15.6	66	15.5	114	999	480	121.4	3086	2909	5.75	237.45		1.54				
	DDH-07	13.4	181	8.21	1538	4.28	11.1	15.1	80	12.1	93	611	262	121.4	1851	1729	6.60	143.66		1.40				
	DDH-08	14.0	246	8.31	2308	5.01	10.4	15.3	84	20.8	96	876	419	111.3	2707	2506	7.44	205.21		1.25				
	DDH-09	13.5	237	8.11	1964	5.32	17.3	25.1	152	19.4	170	718	434	122.1	2495	2317	7.13	188.07	0.36	1.90	0.711176	-8.24	6.21	-7.87
	DDH-10	15.9	177	8.09	1474	3.46	11.6	19.9	29	54.2	72	728	63	135.3	1708	1567	8.25	137.03		0.73				
	DDH-13	7.6	308	8.31	2175	5.21	15.6	114.9	207	26.4	102	856	457	97.5	2753	2724	1.03	214.92		1.70				
	DDH-14	12.7	280	8.09	2343	11.03	11.0	14.3	198	26.2	118	804	600	99.8	2951	2775	5.94	219.93		1.64				
	DDH-15	13.5	173	8.03	1408	3.71	8.7	17.5	81	39.0	64	669	156	95.9	1753	1600	8.72	134.49		0.79				
	DDH-16	10.2	221	8.12	1671	4.16	19.8	35.3	84	23.5	136	662	293	122.1	2070	1898	8.29	157.99		1.73				
	DDH-17		45	7.63	588	1.87	5.4	2.6	56	8.6	34	242	63	51.6	653	709	-8.67	57.06	0.06	0.34	0.713305	-10.4	2.96	
Yalong	YLJ-01	13.1	394	8.28	3427	5.35	238.7	19.8	208	32.8	376	1526	542	109.5	4545	4106	9.65	329.61	0.33	2.70	0.711532	-8.89	5.57	-2.73
	YLJ-10	12.0	211	8.11	2532	6.24	46.2	29.5	229	25.7	202	998	529	143.8	3282	3071	6.43	247.15	0.57	2.04	0.712162	-12.3	2.10	-5.74
	YLJ-15	13.8	315	8.25	2450	6.93	39.7	27.7	227	26.1	194	947	531	147.4	3177	2977	6.29	239.62		1.93				
	YLJ-24	15.7	255	8.43	2559	4.95	32.9	6.0	206	24.8	183	843	502	99.5	2899	3016	-4.05	234.72	0.75	0.74	0.712442	-10.5	2.02	8.34
	YLJ-35	19.8	212	7.56	1975	5.29	26.3	3.4	157	34.8	145	690	342	102.4	2244	2325	-3.60	183.63	0.14	1.86	0.711274	-9.91	-0.28	8.90
	YLJ-36	20.8	205	7.54	1910	5.72	28.7	4.4	146	32.8	147	672	311	107.4	2146	2241	-4.46	177.54	0.06	2.22	0.710435	-9.91	0.18	3.62
	YLJ-39	20.9	195	7.61	1785	7.12	35.0	6.0	142	32.2	148	641	294	111.7	2050	2118	-3.33	168.52	0.15	2.11	0.710519	-11.9	1.43	3.09
tributary	YLJ-02	9.4	203	8.10	2300	3.04	16.6	48.4	151	15.5	96	885	468	124.5	2817	2671	5.20	215.62		1.20				
	YLJ-03	10.5	166	7.86	1373	15.73	14.1	30.3	86	17.1	92	563	243	115.2	1723	1604	6.86	132.84		0.78				
	YLJ-04	10.8	203	8.48	2899	4.34	58.9	32.6	267	27.1	237	1158	595	136.0	3771	3528	6.43	282.20	0.12	2.45	0.711770	-8.97	2.62	-1.87
	YLJ-05	13.7	335	8.16	3025	4.33	57.1	14.6	264	27.7	239	1197	597	138.1	3854	3629	5.83	290.22		2.55				
	YLJ-06	11.8	52	7.89	305	23.57	7.2	14.6	17	5.0	28	154	29	36.6	399	384	3.65	31.73		0.14				
	YLJ-07	8.8	123	7.57	582	4.23	9.1	18.6	205	16.2	65	420	91	58.0	1103	1023	7.19	81.37		0.50				
	YLJ-08	11.2	140	8.01	1089	16.82	10.7	16.2	82	14.9	73	444	179	123.1	1333	1296	2.78	107.76	0.15	0.73	0.714531	-7.32	-1.26	-5.68
	YLJ-09	12.0	211	8.11	1624	2.54	12.8	15.0	192	22.3	120	626	346	139.6	2087	2038	2.33	164.42		1.38				
	YLJ-11	10.1	432	8.41	2663	4.94	9.6	13.0	816	13.8	113	972	1165	128.3	4400	4323	1.75	320.13		2.95				
	YLJ-12	11.9	229	8.28	1687	4.81	9.9	18.1	194	15.7	94	537	525	114.4	2234	2108	5.65	167.01		0.92				
	YLJ-13	12.6	169	8.29	1136	4.50	10.2	10.8	171	20.1	100	387	359	122.9	1611	1504	6.65	121.53		0.83				
	YLJ-14	11.6	251	8.34	2033	5.09	9.4	12.3	161	18.4	103	640	558	152.4	2517	2382	5.35	192.13		1.33				
	YLJ-16	5.8	31	8.00	910	1.29	2.7	1.0	12	3.8	36	296	138	75.2	907	938	-3.40	77.47		0.35	0.714243	-11.1		
	YLJ-17	13.3	237	8.18	2118	5.18	15.7	27.1	120	20.2	142	796	421	123.7	2597	2405	7.40	196.66	0.49	2.07	0.711190	-8.98	6.53	-8.68
	YLJ-18	13.2	346	8.40	3195	3.50	11.6	14.9	285	13.5	108	1048	880	117.5	3979	3794	4.63	297.10		2.04				
	YLJ-19	9.8	376	8.28	4012	5.18	14.5	160.7	59	26.1	169	1151	929	132.9	4354	4311	0.99	342.54		1.95				
	YLJ-20	13.3	312	8.30	2864	5.89	18.3	21.7	186	26.2	179	1043	606	117.5	3504	3281	6.37	263.33		2.66				
	YLJ-21	12.0	369	8.34	3704	4.94	12.5	14.0	56	23.6	149	1003	920	117.5	4018	3847	4.27	306.62		2.00				
	YLJ-22	13.3	237	8.18	2532	5.34	15.9	22.3	144	22.2	155	900	538	119.8	3053	2863	6.21	231.07	0.72	2.15	0.711642	-11.5	5.97	-9.13
	YLJ-23	14.1	106	8.26	1203	2.44	31.4	18.5	26	13.1	88	346	199	121.0	1191	1308	-9.83	106.74		0.54	0.715701	-7.86		

	YLJ-25	13.8	170	8.74	1785	2.42	6.8	2.6	58	14.2	67	589	294	80.2	1847	1914	-3.62	152.62		2.01	0.714979			
	YLJ-26	14.1	190	8.63	1889	14.43	12.2	1.0	51	24.9	329	504	341	124.5	2043	2019	1.19	165.38		0.85				
	YLJ-27	16.2	64	8.75	757	2.10	10.4	1.4	15	17.2	104	212	98	105.9	741	800	-7.95	68.39	0.06	0.36	0.716179	-12.9	2.63	
	YLJ-28	12.6	8	7.54	178	2.82	2.8	0.7	5	8.7	57	51	8	100.2	183	195	-6.09	21.45	0.11	0.10	0.715637	-8.69		
	YLJ-29	15.1	95	7.69	880	14.23	9.3	1.3	64	17.5	137	301	112	110.9	981	1032	-5.23	85.76	0.06	0.42	0.715574	-10.8	-5.84	3.33
	YLJ-30	15.3	201	8.36	2241	2.19	13.3	2.2	14	23.5	46	926	138	80.9	2198	2286	-4.02	185.98		0.92	0.719848	-12.2		
	YLJ-31	18.7	141	6.95	1467	6.19	7.4	1.2	67	17.9	113	546	164	126.0	1552	1616	-4.13	133.10	0.27	0.91	0.714365	-9.53	-4.88	3.57
	YLJ-32	20.4	207	7.87	2147	3.85	6.3	2.0	91	12.4	126	867	194	171.7	2261	2341	-3.54	193.26		1.40	0.707743	-6.9		
	YLJ-33	17.8	275	7.20	2342	3.13	147.8	31.7	81	13.8	168	995	186	94.5	2544	2686	-5.59	212.38	0.32	2.60	0.707974	-9.66	5.95	7.63
	YLJ-34	16.7	177	7.97	1633	3.57	17.4	3.7	81	9.4	74	701	175	131.7	1834	1820	0.75	150.62		1.94	0.707855	-12		
	YLJ-37	21.7	168	6.97	1180	20.00	109.7	15.7	128	29.3	210	497	166	153.1	1564	1580	-1.01	128.56	0.29	1.79	0.711077	-15.6	8.31	15.44
	YLJ-38	22.2	155	7.11	1198	13.98	78.3	14.4	156	29.9	165	494	204	144.6	1592	1616	-1.54	130.37	0.41	1.46	0.711706	-15		
Jinsha	TTH-01	13.7	1054	7.87	2485	12.49	5841.6	3.9	748	286.9	6051	1107	455	115.2	9462	9839	-3.98	643.68	1.15	3.13	0.710306	-0.83	9.20	12.03
	TTH-13	15.4	792	8.15	2840	8.78	3368.0	30.0	811	92.8	3750	1371	814	95.2	8214	7868	4.21	542.82	1.15	6.52	0.710155	-2.67	7.57	5.12
	JSJ-01	15.7	703	8.20	2816	8.57	2552.5	31.3	757	76.1	2838	1301	764	98.8	7045	6922	1.74	481.94	0.83	5.74	0.710277	-7.82	5.36	2.68
	JSJ-16	19.1	480	8.44	2271	8.22	1687.6	25.4	490	54.8	1832	923	517	96.6	4766	4972	-4.33	346.75	1.17	4.41	0.710576	-9.04	4.75	6.99
	JSJ-18	19.9	520	7.58	2251	7.58	1780.3	2.2	474	53.3	1901	907	493	101.6	4753	4989	-4.97	346.44	1.31	4.33	0.710510	-10	5.08	9.86
	JSJ-24	22.2	459	7.27	2172	7.75	1458.3	6.5	425	52.5	1599	886	447	100.9	4317	4495	-4.13	316.83	0.38	3.55	0.710294	-10.1	5.53	7.42
	JSJ-25	21.4	321	7.48	1950	7.39	655.0	5.5	267	41.5	785	748	362	108.1	3047	3152	-3.44	233.26	0.29	2.62	0.710421	-10.6	4.30	6.16
tributary	TTH-02	15.1	337	6.72	2007	9.97	583.0	4.7	415	58.0	944	786	268	70.9	3111	3435	-10.43	249.69	0.63	2.94	0.709893	-1.48	5.93	5.85
	TTH-03	14.0	2387	7.72	1878	15.74	15044.3	4.6	2438	139.0	15031	1988	916	58.7	20980	21819	-4.00	1339.2	6.76	16.83	0.709740	-8.4	10.24	11.74
	TTH-04	12.9	284	7.86	1571	6.91	858.5	8.2	203	57.0	759	579	380	39.4	2735	2850	-4.21	200.85		2.27				
	TTH-05	15.4	681	7.89	2436	6.36	2774.4	11.1	854	48.1	2260	893	1257	54.4	6609	6936	-4.94	453.21		4.91				
	TTH-06	17.8	400	8.27	2466	4.73	232.7	23.9	347	26.2	466	1207	420	61.6	3747	3422	8.66	267.64	0.65	3.41	0.709125	-4	0.21	1.28
	TTH-07	15.3	377	8.28	3290	5.94	61.9	22.3	83	29.5	251	1408	455	115.2	4005	3546	11.46	293.63		1.93				
	TTH-08	15.3	378	8.13	3586	3.49	242.6	37.2	1086	25.3	472	1495	1472	84.5	6432	6042	6.06	446.60	0.59	4.46	0.712572	-4.89	2.66	-6.28
	TTH-09	10.3	339	8.15	4071	6.61	400.2	33.4	1000	38.2	1007	1780	1109	99.5	6823	6511	4.58	489.60	0.90	7.95	0.711130	-6.24	7.08	0.48
	TTH-10	16.0	1055	8.11	3006	3.99	99.0	45.6	447	35.3	304	1312	745	108.8	4454	4048	9.12	318.25		2.88				
	TTH-11	13.5	641	8.41	3680	5.94	49.2	9.4	336	20.4	276	1570	526	126.7	4489	4418	1.59	349.70	0.33	2.32	0.710462	-6.85	-1.00	-2.54
	TTH-12	13.5	331	8.22	2177	4.20	20.9	24.8	414	20.2	175	1095	504	78.0	3392	3054	9.97	240.49		2.51				
	JSJ-02	11.6	273	7.05	2296	11.00	16.5	21.8	194	32.6	101	939	464	78.8	2940	2734	7.03	218.08		1.32				
	JSJ-03	12.2	242	7.55	1751	2.96	12.5	11.5	284	19.1	57	976	254	82.3	2537	2347	7.47	187.66		1.63				
	JSJ-04	13.2	129	6.92	2035	3.00	13.0	19.2	198	24.9	67	911	387	88.1	2688	2466	8.27	198.57		1.31				
	JSJ-05	15.7	161	7.87	1285	16.04	12.4	19.6	89	20.0	79	540	219	80.9	1616	1512	6.46	123.33		0.82				
	JSJ-06	15.8	174	8.08	1432	4.50	12.5	13.9	95	17.2	98	604	227	97.4	1776	1653	6.96	136.32		0.86				
	JSJ-07	14.0	187	8.00	1396	5.39	10.7	13.4	175	20.2	82	653	243	78.8	1896	1776	6.32	142.79		0.99				
	JSJ-08	13.7	276	8.21	2556	2.95	6.6	5.6	196	17.0	83	1102	372	99.5	3048	2962	2.80	237.07		1.58				
	JSJ-09	14.7	256	7.93	1396	2.79	9.8	14.0	542	10.5	73	763	514	91.6	2638	2507	4.97	189.13		2.06				
	JSJ-10	14.7	279	8.32	2596	4.74	30.8	23.7	141	19.4	141	1186	236	108.1	3005	2938	2.24	238.35		4.18				
	JSJ-11	14.7	220	8.21	1929	3.95	13.9	26.4	150	15.6	116	916	171	116.0	2305	2274	1.35	185.38	0.17	2.17	0.708557	-6.52	1.22	-2.69

	JSJ-12	14.3	175	8.22	2189	4.53	35.0	28.1	239	25.7	254	918	293	98.1	2702	2734	-1.19	216.15		1.56				
	JSJ-13	15.2	310	8.31	2769	6.19	62.5	22.6	149	27.5	290	1213	352	120.2	3446	3159	8.35	259.08		2.64				
	JSJ-14	16.0	226	8.38	2353	4.64	46.9	1.2	91	35.6	147	851	243	90.9	2370	2587	-9.15	204.31	0.63	0.76	0.711584	-12.6	9.93	9.81
	JSJ-15	20.2	320	8.65	2667	17.47	254.8	1.3	314	46.7	662	938	404	192.5	3392	3568	-5.17	278.23		4.08	0.709537	-5		
	JSJ-17	17.9	85	7.16	840	2.01	17.2	15.0	16	23.2	71	304	86	116.7	874	905	-3.58	78.12	0.31	0.91	0.710025	-17.8		
	JSJ-19	13.1	67	8.21	757	2.73	9.5	11.9	25	11.8	18	262	78	125.3	710	832	-17.08	70.53	0.23	0.82	0.708667	-10		
	JSJ-20	8.6	172	8.01	2040	1.17	6.9	2.4	4	8.2	13	753	223	55.2	1973	2059	-4.35	164.77		0.49	0.708679	-13.6		
	JSJ-21	13.2	1	7.38	113	1.65	5.3	5.3	4	8.7	39	36	20	67.3	161	134	16.86	15.06		0.06	0.715488	-9.58		
	JSJ-22	14.0	21	7.69	316	1.71	4.6	7.8	5	9.7	58	85	41	84.5	320	341	-6.74	31.69	0.06	0.14	0.715319	-7.86		
	JSJ-23	16.0	41	8.00	525	1.38	2.7	0.8	7	12.6	42	181	48	95.2	514	544	-5.93	48.50	0.05	0.22	0.716002	-10.6		
Lancang	LCJ-01	13.4	572	8.06	2745	9.35	850.6	34.0	1133	57.1	1206	1831	725	83.0	6374	5905	7.35	434.61	1.95	7.76	0.708682	0.166	-0.46	4.59
	LCJ-11	7.7	516	8.19	2698	8.29	339.5	34.8	713	37.9	557	1415	636	90.2	4697	4506	4.07	339.21	0.61	4.92	0.709325	-7.77	2.60	3.15
	LCJ-14	15.6	364	8.40	2356	7.06	295.6	5.6	654	72.4	510	1123	501	85.2	3830	3972	-3.71	294.31	2.59	4.74	0.709427	-11	4.21	12.27
	LCJ-17	17.4	370	7.36	2315	7.35	323.5	5.8	603	42.4	505	1083	484	88.1	3682	3859	-4.81	284.87	2.26	4.12	0.709614	-10.5	3.52	9.74
	LCJ-20	20.0	322	7.40	2033	6.84	245.8	3.9	516	38.1	404	977	418	88.8	3232	3322	-2.80	248.10	1.36	3.66	0.709788	-9.6	2.98	6.10
tributary	LCJ-02	11.9	354	8.12	2828	6.22	35.1	81.7	387	29.8	253	1364	528	82.3	4067	3726	8.39	295.54		2.97				
	LCJ-03	12.4	316	8.32	2627	4.44	16.2	27.3	297	34.6	225	1190	443	70.2	3525	3269	7.27	260.30		1.53				
	LCJ-04	11.5	281	8.16	2402	4.42	15.8	18.6	390	24.3	163	1045	417	87.3	3111	3221	-3.54	247.71		1.97				
	LCJ-05	6.8	521	8.20	2674	7.93	420.4	32.7	629	37.1	642	1413	576	88.1	4655	4393	5.63	332.66	0.70	5.09	0.708924	-10.7	3.69	5.86
	LCJ-06	12.1	366	8.10	2970	1.14	56.1	16.6	770	22.7	176	1610	426	98.8	4271	4585	-7.35	343.92	0.50	3.74	0.708781	-7.95	4.81	4.50
	LCJ-07	12.4	383	8.10	2189	8.75	128.1	25.7	766	40.1	301	1274	672	66.6	4234	3884	8.25	293.30	2.86	3.23	0.710431	-5.54	-0.52	0.01
	LCJ-08	7.5	311	8.25	2568	9.80	200.2	30.4	880	39.2	403	1418	756	81.6	4789	4568	4.61	341.18	0.84	4.56	0.709989	-4.81	0.36	-4.12
	LCJ-09	12.3	199	8.00	1006	3.21	12.3	18.8	392	18.3	69	659	307	63.7	2020	1825	9.66	140.69		0.91				
	LCJ-10	11.2	338	8.27	2615	4.53	18.1	19.7	283	25.4	107	1033	685	93.8	3569	3223	9.67	255.78		2.13				
	LCJ-12	8.5	308	8.16	1644	4.74	49.9	21.4	476	23.0	125	853	456	70.9	2766	2673	3.39	202.52		1.96				
	LCJ-13	17.0	285	8.33	1964	5.82	17.2	28.4	301	26.0	188	910	389	88.8	2811	2619	6.84	207.81		3.55				
	LCJ-15	5.6	133	8.25	927	3.43	7.4	4.2	235	11.2	57	418	267	60.2	1439	1411	1.99	108.25		0.12	0.727501	-8.45		
	LCJ-16	15.9	179	8.46	2019	4.35	11.0	3.7	42	41.3	92	581	418	95.2	2131	2122	0.42	170.79		2.53	0.713219	-7.66		
	LCJ-18	14.6	259	7.33	1944	0.89	3.4	1.5	373	8.0	56	710	526	70.2	2535	2695	-6.30	201.60	0.20	2.61	0.714443	-3.56	-6.15	-4.56
	LCJ-19	15.8	160	7.37	1231	5.39	8.6	18.3	249	13.1	93	597	192	76.6	1685	1760	-4.48	136.35	0.32	1.13	0.715992	-5.68	2.78	-2.23
Nu	NJ-01	12.9	425	7.99	3458	17.27	235.7	1.5	438	93.6	1442	720	709	123.1	4395	4588	-4.41	352.07	0.26	9.20		-3.61	8.31	14.84
	NJ-23	17.2	254	7.18	1672	6.33	21.9	2.0	504	25.9	161	721	485	64.4	2599	2711	-4.32	200.70	1.60	1.46	0.713104	-9.97	1.73	2.47
	NJ-26	20.0	215	7.28	1452	6.50	18.3	4.9	354	28.4	129	612	363	76.6	2108	2189	-3.82	165.63	1.03	1.15	0.713933	-9.84	1.05	-6.95
	NJ-27	20.5	236	7.35	1424	6.42	17.1	4.6	341	30.8	125	602	354	75.9	2068	2134	-3.21	161.97	1.02	1.14	0.714014	-10.9	1.15	-5.93
tributary	NJ-02	7.7	245	8.14	1598	7.96	11.9	78.7	445	17.1	144	848	426	68.0	2709	2585	4.56	197.98		0.75				
	NJ-03	11.5	397	8.22	3623	10.83	45.2	84.7	583	41.0	341	1394	762	104.5	4694	4929	-5.01	374.12	1.44	2.10	0.711226	-5.86	2.33	-3.27
	NJ-04	4.0	280	8.09	1574	7.23	11.8	29.0	564	18.7	142	934	539	65.2	3106	2750	11.48	210.92		1.29				
	NJ-05	9.9	298	8.32	2201	3.56	16.0	18.3	304	20.8	93	953	538	63.7	3094	2847	7.97	223.25		1.30				
	NJ-06	10.0	276	8.25	2095	3.53	15.8	25.9	309	21.4	92	952	531	60.9	3079	2759	10.41	217.36		1.32				
	NJ-07	11.6	252	8.14	1574	5.94	10.9	20.9	1057	34.3	137	999	815	73.0	3799	3726	1.90	268.03		3.22				

	NJ-08	11.5	249	8.12	1787	2.96	11.1	24.3	310	25.7	85	914	395	66.6	2727	2446	10.32	193.90		1.07				
	NJ-09	10.7	330	8.19	2296	5.44	33.7	40.2	506	31.6	221	1104	658	72.3	3776	3387	10.28	263.30		2.01				
	NJ-10	9.9	174	8.00	1361	2.88	8.7	20.5	137	26.4	52	682	162	58.7	1768	1668	5.67	134.88		0.66				
	NJ-11	9.6	204	8.08	1304	3.16	11.5	20.0	242	24.2	70	731	275	58.0	2106	1822	13.49	146.49		1.22				
	NJ-12	8.1	196	8.10	1657	3.64	7.9	17.7	155	21.1	62	758	332	76.6	2262	1995	11.77	162.63		1.08				
	NJ-13	8.8	265	8.08	1811	4.59	11.3	20.5	384	30.1	104	740	638	74.5	2891	2616	9.53	202.31		1.10				
	NJ-14	8.8	264	8.17	1680	3.96	12.6	103.7	283	25.0	108	692	572	85.9	2660	2367	11.02	186.86		1.14				
	NJ-15	8.7	441	8.24	2130	5.07	10.7	24.5	1033	38.7	96	1251	968	75.9	4573	4236	7.36	313.02		3.28				
	NJ-16	7.6	314	7.82	1728	4.30	18.0	19.6	685	27.4	102	996	646	47.3	3413	3139	8.03	234.94		2.26				
	NJ-17	7.1	174	8.04	1527	4.66	15.8	16.7	507	24.7	96	706	638	52.3	2809	2577	8.25	193.55		1.30				
	NJ-18	10.1	322	8.36	2544	2.96	11.5	20.3	248	16.7	58	1083	585	71.6	3410	3075	9.81	244.63		1.24				
	NJ-19	9.0	375	8.19	2876	6.16	31.9	20.8	509	24.0	156	1139	929	98.8	4317	3951	8.46	305.46	0.72	2.97	0.709619	-5.59	-5.50	-2.04
	NJ-20	9.6	91	8.05	661	2.12	2.9	1.6	181	13.8	38	366	99	48.71	981	1030	-4.99	79.39		0.65				
	NJ-21	10.8	71	7.89	657	4.19	9.0	1.2	75	15.2	74	277	69	43.7	780	821	-5.22	65.38	0.16	0.37	0.726912	-3.7	-1.55	
	NJ-22	15.4	138	7.44	1002	8.91	17.1	1.8	177	22.9	135	457	119	63.0	1310	1384	-5.62	108.02	0.70	0.74	0.718816	-9.31	-0.70	-2.51
	NJ-24	15.2	165	8.37	1235	9.85	12.8	13.8	150	17.1	93	559	240	65.2	1708	1571	8.03	126.19		0.64				
	NJ-25	15.1	69	7.35	1218	8.94	8.6	18.2	118	16.2	83	460	193	66.6	1407	1490	-5.92	116.96	0.13	0.60	0.716951	-8.7	-0.59	0.61
Yarlung-	YLZB-07	16.9	193	8.03	1077	9.54	62.2	91.0	325.7	27.2	208	708	181.6	98.1	2015	1891	6.17	149.52		1.30				
Tsangpo	YLZB-08	17.7	188	7.85	1385	8.94	55.9	13.6	612.9	27.1	203	1105	180.4	106.7	2801	2689	3.99	207.08		1.42				
	YLZB-10	17.5	188	7.90	1030	8.61	51.9	25.3	509.1	26.6	197	686	184.8	93.1	1964	2134	-8.64	158.37	1.11	1.24	0.712764	-11.1	-2.87	-8.59
	YLZB-13	17.2	169	7.95	935	7.74	45.8	25.1	273.8	22.6	151	578	154.7	87.3	1640	1561	4.80	123.18	0.78	1.12	0.712484	-4.42	-2.71	-8.40
	YLZB-15	16.2	164	7.84	864	7.26	40.1	24.9	257.5	22.3	143	560	148.1	83.0	1580	1451	8.17	115.69	0.87	1.05	0.712469	-12.8	-2.72	-8.96
tributary	YLZB-01	11.4	197	7.91	1668	18.18	70.6	3.0	170.1	22.3	393	671	125.9	126.0	2010	2100	-4.50	168.54		1.29				
	YLZB-02	18.0	255	7.83	1669	4.83	48.9	4.0	324.1	33.1	267	699	337.5	88.8	2374	2375	-0.04	183.99		1.33				
	YLZB-03	13.7	84	7.48	531	4.15	29.8	3.6	122.7	12.0	143	234	78.4	106.7	779	814	-4.49	66.97	0.07	0.95	0.707401	-6.31	0.10	1.51
	YLZB-04	12.6	53	6.50	471	1.89	7.9	3.9	95.9	15.5	54	222	76.9	95.2	667	677	-1.40	56.83	0.07	0.35	0.710130	-8.12	0.76	5.99
	YLZB-05	13.4	139	8.14	989	7.28	31.4	4.3	214.2	17.3	113	484	154.5	72.3	1408	1460	-3.70	113.18	0.15	0.88	0.719797	-9.9	-1.93	5.17
	YLZB-06	15.1	69	8.00	497	17.17	115.7	3.4	55.4	32.9	171	224	27.4	70.9	705	744	-5.53	59.37		0.41				
	YLZB-9	11.2	89	7.49	391	2.72	9.6	22.6	133.7	20.4	55	334	56.4	85.9	856	693	19.01	60.41		0.51				
	YLZB-11	13.2	292	7.93	911	3.87	9.5	14.0	969.0	11.4	97	1033	458.3	63.7	3092	2877	6.95	208.94	1.10	2.64	0.714340	-10.2	0.94	-8.72
	YLZB-12	11.8	33	6.91	178	0.00	1.9	3.7	20.5	17.0	22	78	13.9	46.6	223	224	-0.66	20.52		0.12				
	YLZB-14	10.9	66	7.00	296	2.23	6.7	10.2	89.6	10.8	21	198	51.9	50.9	531	494	6.97	40.70	0.18	0.26	0.711805	-8.68	5.81	-2.26
	YLZB-16	10.2	176	7.84	497	3.72	5.0	3.6	662.3	10.5	83	596	223.4	62.3	1733	1834	-5.85	129.76		1.33	0.718808	-2.4		
	YLZB-17	9.2	238	8.44	1787	1.63	5.8	4.1	427.2	22.8	93	714	529.7	46.6	2603	2653	-1.91	197.82		2.57	0.717670	-4.6		
	YLZB-18	12.1	132	8.16	792	3.32	13.9	2.9	300.1	13.7	74	472	163.7	63.0	1360	1412	-3.88	106.79	0.12	1.10	0.714144	-3.56	-0.66	-5.72
	YLZB-19				678	3.54	11.2	2.8	242.5	15.2	55	378	146.3	59.4	1118	1181	-5.55	89.40		0.77				
	YLZB-20				586	5.59	14.4	1.7	126.0	16.6	37	305	65.7	50.1	795	860	-8.25	66.91		0.45				
	YLZB-21	12.4	10	8.08	177	1.31	4.0	1.0	11.0	11.1	50	57	13.0	93.1	200	205	-2.56	21.83		0.16	0.712457	-6.41		
	YLZB-22	12.6	44	7.39	441	3.27	5.0	1.3	77.8	22.5	26	231	27.7	64.4	566	606	-7.12	49.94	0.05	0.27	0.715847	-5.32	-2.09	4.32
	YLZB-23	14.4	177	7.28	1353	3.17	3.5	1.4	231.3	103.9	54	698	102.1	121.7	1758	1823	-3.73	148.03		1.17				

YLZB-24	9.8	71	8.02	654	18.37	2.5	1.5	75.7	13.6	30	318	48.7	33.0	777	828	-6.60	64.84		0.30				
YLZB-25	11.0	47	8.14	600	4.57	3.1	1.1	13.3	16.1	41	236	35.9	73.7	602	635	-5.63	54.48		0.25				
YLZB-26	11.6	88	7.23	803	6.22	3.5	1.4	102.2	15.1	63	348	116.2	28.7	1007	1019	-1.20	79.69	0.16	0.77	0.720015	-7.21	2.47	0.75
YLZB-27	10.9	123	8.16	1070	11.23	4.3	1.6	129.0	10.7	35	421	195.8	47.3	1280	1345	-5.11	103.82		0.87				
YLZB-28	9.5	31	8.03	384	2.61	3.0	1.3	31.4	10.6	21	168	26.1	52.3	420	454	-8.18	38.09		0.16				
YLZB-29	11.9	90	8.14	663	6.70	4.8	1.4	144.3	26.4	27	367	84.5	43.0	955	964	-0.94	75.65		0.69				
YLZB-30	11.9	94	7.43	870	6.41	6.3	1.4	127.5	23.1	26	406	105.0	36.6	1070	1139	-6.42	88.25	0.25	1.16	0.714726	-5.4	4.20	5.52
YLZB-31	10.5	142	7.85	781	9.83	12.0	12.7	213.0	35.0	45	480	144.6	41.6	1328	1242	6.52	97.12		0.61				

Table S2. Chemical compositions of precipitation at different sites located within the studied area (in µmol l^{-1} and molar ratio).

River Basins	Sites	Cl^- µmol L^{-1}	SO_4^{2-} µmol L^{-1}	Na^+ µmol L^{-1}	K^+ µmol L^{-1}	Mg^{2+} µmol L^{-1}	Ca^{2+} µmol L^{-1}	SO4/Cl	Na/Cl	K/Cl	Mg/Cl	Ca/Cl	References
Upper Huang	Madoi	28.17	19.79	17.39	10.26	20.58	155	0.70	0.62	0.36	0.73	5.50	ref. 15
	Henan	39.44	45.83	30.43	15.38	28.81	110	1.16	0.77	0.39	0.73	2.79	
	Xinghai	16.9	21.88	8.7	10.26	8.23	70	1.29	0.51	0.61	0.49	4.14	
	Darlag	19.72	12.5	13.04	10.26	4.12	37.5	0.63	0.66	0.52	0.21	1.90	
	Waliguan	6.1	12	8.7	3.8	6.05	17	1.97	1.43	0.62	0.99	2.79	ref. 16
Dadu	Gongga	6.15	16.33	3.41	1.94	1.99	12.91	2.66	0.55	0.32	0.32	2.10	ref. 17
Yalong	Lijiang	39.5	131.4	27.0	14.6	9.5	90.0	3.33	0.68	0.37	0.24	2.28	ref. 18
Jinsha	Dongkemadi	1.0	2.15	4.1	0.6	0.15	2.7	2.15	4.10	0.60	0.15	2.70	ref. 19
Lancang	Lijiang	11.56	16.32	2.54		3.87	25.1	1.41	0.22		0.33	2.17	ref. 20
Nu	Panzhihua	19.42	68.1	9.64	5.41	7.35	49	3.51	0.50	0.28	0.38	2.52	ref. 21
Yarlung-Tsangpo	Nam Co	19.17	7.75	15.44	14.49	3.72	32.79	0.40	0.81	0.76	0.19	1.71	ref. 22
	Nam Co	1.1	1.95	1.9	0.4	0.45	3.95	1.77	1.73	0.36	0.41	3.59	ref. 23
	Qomolangma	27.1	1.6	26	4.5	0.85	26.7	0.06	0.96	0.17	0.03	0.99	
	Lulang	6.74	1.31	6.73	1.88	0.83	17.02	0.19	1.00	0.28	0.12	2.53	ref. 24
	Sejila Mount.	40.1	9.7	58.5	13.1	14.4	61.5	0.24	1.46	0.33	0.36	1.53	ref. 25

Table S3. Calculated contributions (in %) from different reservoirs to the total cationic loads (the sum of Na^+, K^+, Ca^{2+} and Mg^{2+}) and riverine sulfate (SO_4^{2-}) for the studied rivers.

River	Sample number	Contribution to cationic load				Contribution to sulfate		
		Atmosphere	Evaporite	Silicate	Carbonate	f_{rain}	f_{pryite}	f_{gypsum}
		%	%	%	%	%	%	%
Upper Huang	HH-01	1.0	55.9	7.3	35.8	3.3	34.4	62.3
	HH-06	2.0	24.2	14.6	59.2	2.4	40.6	57.0
	HH-14	3.6	13.7	21.8	60.9	4.7	25.0	70.3
	HH-15	2.5	14.4	17.0	66.1	3.7	59.9	36.5
	HH-23	1.8	16.5	16.9	64.7	3.3	77.4	19.3
	HH-26	1.9	6.0	15.7	76.4			
tributary	HH-02	1.8	22.8	15.8	59.6	7.7	32.0	60.2
	HH-03	1.7	10.9	2.2	85.1			
	HH-04	1.3	5.1	32.1	61.5			
	HH-05	2.8	1.2	18.1	77.8			
	HH-07	1.7	3.5	17.4	77.4			
	HH-08	1.8	8.5	18.9	70.7	1.9	74.0	24.1
	HH-09	6.6	2.6	27.3	63.5			
	HH-10	3.8	1.6	23.6	71.1			
	HH-11	6.0	3.5	29.0	61.5			
	HH-12	3.0	0.0	8.2	88.9			
	HH-13	5.7	2.3	26.9	65.1			
	HH-16	1.8	2.0	9.9	86.3			
	HH-17	1.4	1.7	11.2	85.7			
	HH-18	1.7	7.9	12.1	78.3			
	HH-19	1.6	16.4	19.2	62.8	4.4	63.0	32.6
	HH-20	1.6	10.4	17.5	70.4			
	HH-21	1.3	4.1	36.6	58.1			
	HH-22	1.8	17.2	29.2	51.9	2.3	48.2	49.5
	HH-24	2.1	2.3	9.3	86.3			
	HH-25	2.3	6.5	15.6	75.6			
Dadu	DDH-11	1.9	0.6	13.9	83.6			
	DDH-12	1.3	0.8	16.3	81.6			
	DDH-18	1.6	6.2	18.4	73.8	12.1	41.6	46.3
	DDH-19	1.7	6.5	19.2	72.6	10.8	47.9	41.3
tributary	DDH-01	2.5	3.0	26.5	68.1			
	DDH-02	1.7	1.8	17.2	79.3			
	DDH-03	1.5	6.5	16.4	75.6	11.9	35.1	53.0
	DDH-04	1.8	1.4	20.7	76.1			
	DDH-05	1.0	0.7	14.4	83.9			
	DDH-06	1.1	0.5	11.1	87.4			
	DDH-07	1.8	0.6	14.8	82.8			
	DDH-08	1.3	0.4	11.2	87.2			
	DDH-09	1.3	6.7	19.7	72.2	9.4	39.3	51.3
	DDH-10	1.9	0.7	16.6	80.8			
	DDH-13	1.2	0.7	11.5	86.6			
	DDH-14	1.1	0.4	12.8	85.7			
	DDH-15	1.9	0.4	13.9	83.8			

	DDH-16	1.6	1.3	18.9	78.2			
	DDH-17	5.1	5.0	16.0	73.9	25.7	43.5	30.9
Yalong	YLJ-01	0.4	13.8	10.2	75.6	4.3	44.5	51.2
	YLJ-10	0.5	7.4	15.6	76.4	3.9	58.3	37.8
	YLJ-15	0.6	2.2	16.0	81.2			
	YLJ-24	0.6	6.9	17.0	75.5	4.4	58.4	37.2
	YLJ-35	0.8	5.5	18.5	75.2	5.7	66.7	27.6
	YLJ-36	0.8	5.9	19.0	74.2	6.1	64.7	29.2
	YLJ-39	0.9	7.2	19.1	72.8	6.3	59.7	34.0
tributary	YLJ-02	0.7	1.0	9.7	88.7			
	YLJ-03	1.1	1.2	15.5	82.2			
	YLJ-04	0.5	8.1	15.4	76.0	3.4	56.6	40.1
	YLJ-05	0.5	2.6	15.4	81.5			
	YLJ-06	4.5	2.1	17.6	75.9			
	YLJ-07	1.6	1.1	17.9	79.3			
	YLJ-08	1.4	3.6	16.2	78.9	11.0	67.8	21.2
	YLJ-09	0.9	0.9	17.4	80.8			
	YLJ-11	0.4	0.3	7.9	91.4			
	YLJ-12	0.8	0.6	12.7	85.8			
	YLJ-13	1.1	0.9	18.9	79.1			
	YLJ-14	0.7	0.5	12.7	86.0			
	YLJ-16	2.0	0.0	11.9	86.0			
	YLJ-17	0.7	5.6	16.1	77.6	7.5	39.0	53.4
	YLJ-18	0.5	0.4	8.2	90.9			
	YLJ-19	0.4	0.5	11.9	87.1			
	YLJ-20	0.5	0.8	15.2	83.4			
	YLJ-21	0.5	0.5	11.5	87.6			
	YLJ-22	0.6	5.4	15.1	78.9	6.3	41.9	51.8
	YLJ-23	1.5	4.4	16.0	78.1			
	YLJ-25	1.0	0.4	11.5	87.1			
	YLJ-26	0.8	0.8	44.1	54.3			
	YLJ-27	2.2	2.2	38.6	56.9	61.8	25.6	12.6
	YLJ-28	7.8	0.1	76.0	16.1			
	YLJ-29	1.7	1.4	39.0	57.9	14.1	84.1	1.8
	YLJ-30	0.9	0.9	6.7	91.5			
	YLJ-31	1.1	1.0	22.0	75.8	13.4	80.6	5.9
	YLJ-32	0.8	0.3	16.9	82.0			
	YLJ-33	0.7	13.6	3.4	82.3	11.1	39.4	49.5
	YLJ-34	1.0	1.5	10.4	87.1			
	YLJ-37	1.1	20.4	21.0	57.5	7.0	32.3	60.7
	YLJ-38	1.1	8.5	18.9	71.6			
Jinsha	TTH-01	0.3	80.3	7.6	11.8	0.8	32.1	67.1
	TTH-13	0.3	64.0	11.4	24.3	0.7	38.6	60.7
	JSJ-01	0.4	59.1	10.3	30.1	0.8	47.2	52.0
	JSJ-16	0.6	57.9	8.1	33.4	1.2	49.4	49.5
	JSJ-18	0.6	60.2	6.8	32.4	1.2	48.0	50.7
	JSJ-24	0.7	56.2	8.8	34.3	1.4	46.2	52.4
	JSJ-25	1.1	40.2	12.1	46.7	2.2	50.6	47.2
tributary	TTH-02	1.0	39.1	30.0	29.9	1.4	44.6	54.0

	TTH-03	0.1	92.9	0.6	6.5	0.2	28.3	71.4
	TTH-04	1.1	48.2	0.0	57.2			
	TTH-05	0.5	62.2	0.0	55.0			
	TTH-06	1.0	16.0	18.1	65.0	1.7	66.9	31.4
	TTH-07	1.0	2.8	14.7	81.6			
	TTH-08	0.6	19.9	10.8	68.6	0.5	57.9	41.5
	TTH-09	0.5	25.1	25.1	49.3	0.6	40.5	58.9
	TTH-10	0.9	4.0	14.5	80.7			
	TTH-11	0.9	5.7	15.3	78.2	1.7	71.7	26.6
	TTH-12	1.1	1.0	14.0	83.9			
	JSJ-02	1.3	0.9	9.9	87.8			
	JSJ-03	1.6	0.7	5.8	91.9			
	JSJ-04	1.5	0.7	6.9	90.9			
	JSJ-05	2.4	1.1	12.9	83.6			
	JSJ-06	2.2	1.0	14.6	82.2			
	JSJ-07	2.0	0.8	11.8	85.3			
	JSJ-08	1.3	0.2	7.8	90.6			
	JSJ-09	1.5	0.5	7.1	90.9			
	JSJ-10	1.3	1.8	11.4	85.6			
	JSJ-11	1.7	5.2	13.4	79.8	3.9	61.8	34.3
	JSJ-12	1.4	2.2	24.1	72.4			
	JSJ-13	1.1	3.2	19.9	75.8			
	JSJ-14	1.6	8.3	14.1	76.1	6.4	26.3	67.3
	JSJ-15	1.0	12.3	33.1	53.6			
	JSJ-17	4.2	3.0	19.4	73.4			
	JSJ-19	5.5	1.8	2.8	89.8			
	JSJ-20	2.0	0.4	0.4	97.2			
	JSJ-21	19.6	2.5	45.7	32.2			
	JSJ-22	10.5	1.0	41.5	47.0			
	JSJ-23	7.2	0.0	21.2	71.6			
Lancang	LCJ-01	0.2	30.9	16.4	52.5	0.4	70.2	29.4
	LCJ-11	0.3	23.8	14.7	61.2	0.7	58.1	41.2
	LCJ-14	0.4	27.3	18.9	53.3	0.7	51.7	47.5
	LCJ-17	0.4	27.9	15.9	55.8	0.8	54.4	44.8
	LCJ-20	0.5	25.2	16.0	58.3	0.9	56.4	42.6
tributary	LCJ-02	0.4	1.5	17.5	80.6			
	LCJ-03	0.5	0.7	19.6	79.3			
	LCJ-04	0.6	0.8	15.9	82.8			
	LCJ-05	0.3	26.4	14.7	58.6	0.8	53.8	45.5
	LCJ-06	0.4	19.6	9.7	70.3	0.6	49.4	50.0
	LCJ-07	0.4	15.2	14.0	70.4	0.6	70.3	29.0
	LCJ-08	0.4	18.5	14.0	67.2	0.5	66.9	32.5
	LCJ-09	0.9	0.8	10.7	87.6			
	LCJ-10	0.5	0.8	9.3	89.4			
	LCJ-12	0.6	3.2	10.1	86.1			
	LCJ-13	0.6	0.9	19.9	78.6			
	LCJ-15	1.2	0.5	12.6	85.7			
	LCJ-16	0.8	0.7	15.4	83.1			
	LCJ-18	0.7	1.9	7.3	90.0	1.3	92.1	6.6

	LCJ-19	1.0	12.1	16.9	70.0	1.9	56.7	41.4
Nu	NJ-01	0.4	17.1	67.9	14.6	2.3	34.8	62.9
	NJ-23	0.8	14.8	17.9	66.5	2.0	60.8	37.2
	NJ-26	0.9	12.0	18.1	68.9	2.9	63.0	34.2
	NJ-27	1.0	11.9	18.4	68.8	3.0	62.5	34.5
tributary	NJ-02	0.7	0.6	15.9	82.7			
	NJ-03	0.4	10.8	20.2	68.6	1.7	58.6	39.7
	NJ-04	0.7	0.5	13.9	84.9			
	NJ-05	0.7	0.8	9.0	89.5			
	NJ-06	0.7	0.8	9.0	89.5			
	NJ-07	0.5	0.4	11.9	87.1			
	NJ-08	0.8	0.6	10.2	88.5			
	NJ-09	0.5	1.5	16.4	81.5			
	NJ-10	1.2	0.6	10.5	87.7			
	NJ-11	1.0	0.8	10.8	87.5			
	NJ-12	0.9	0.4	9.2	89.4			
	NJ-13	0.7	0.6	11.8	86.9			
	NJ-14	0.8	0.7	12.8	85.8			
	NJ-15	0.5	0.3	7.5	91.8			
	NJ-16	0.6	0.9	9.1	89.4			
	NJ-17	0.7	0.9	10.5	87.9			
	NJ-18	0.6	0.5	5.3	93.6			
	NJ-19	0.5	3.3	9.9	86.3	2.0	89.2	8.8
	NJ-20	2.1	0.0	13.8	84.1			
	NJ-21	2.5	4.7	27.6	65.3	13.6	67.5	18.9
	NJ-22	1.5	8.2	28.9	61.5	5.8	68.3	25.9
	NJ-24	1.2	1.1	16.2	81.6			
	NJ-25	1.4	4.7	18.1	75.8	8.6	66.4	25.0
Yarlung-	YLZB-07	0.7	5.4	22.9	71.1			
Tsangpo	YLZB-08	0.5	3.6	17.1	78.8			
	YLZB-10	0.7	13.9	23.4	62.1	0.2	79.8	20.0
	YLZB-13	0.8	11.0	20.9	67.3	0.4	79.1	20.6
	YLZB-15	0.8	10.4	21.1	67.6	0.4	79.1	20.5
tributary	YLZB-01	0.6	5.7	44.0	49.7			
	YLZB-02	0.5	3.5	28.3	67.6			
	YLZB-03	1.6	14.2	40.8	43.4	0.8	67.8	31.4
	YLZB-04	2.0	10.5	23.5	64.0	1.1	65.1	33.9
	YLZB-05	1.0	10.4	19.0	69.6	0.5	75.9	23.6
	YLZB-06	1.6	25.0	26.2	47.2			
	YLZB-9	1.6	1.7	19.6	77.2			
	YLZB-11	0.5	21.7	9.4	68.5	0.1	64.9	35.0
	YLZB-12	5.6	0.0	36.9	57.5			
	YLZB-14	2.6	18.8	11.6	67.1	1.1	45.3	53.6
	YLZB-16	0.8	0.3	14.5	84.3			
	YLZB-17	0.5	0.3	11.8	87.4			
	YLZB-18	1.0	13.5	14.9	70.6	0.3	71.0	28.6
	YLZB-19	1.2	1.6	14.1	83.1			
	YLZB-20	1.7	2.9	12.2	83.1			
	YLZB-21	5.6	1.6	63.4	29.4			

YLZB-22	2.4	6.7	17.7	73.2	1.3	76.1	22.6
YLZB-23	0.8	0.2	19.0	80.0			
YLZB-24	1.8	0.1	13.8	84.3			
YLZB-25	2.2	0.4	22.7	74.7			
YLZB-26	1.4	7.9	20.0	70.7	1.0	58.4	40.6
YLZB-27	1.1	0.4	8.8	89.8			
YLZB-28	3.3	0.5	16.8	79.4			
YLZB-29	1.5	0.6	12.0	86.0			
YLZB-30	1.3	11.6	9.4	77.7	0.8	51.7	47.5
YLZB-31	1.0	1.4	12.2	85.3			

Supplementary References

1. Zhang, Y., Li, B. & Zheng, D. A discussion on the boundary and area of the Tibetan Plateau in China. *Geogr. Res.* **21**, 1-8 (2002).

2. Yao, T. D. et al. Different glacier status with atmospheric circulations in Tibetan Plateau and surroundings. *Nat. Clim. Chang.* **2**, 663-667 (2012).

3. Yang, M., et al. Permafrost degradation and its environmental effects on the Tibetan Plateau: A review of recent research. *Earth-Sci. Rev.* **103**, 31-44 (2010).

4. Galy, A. & France-Lanord, C. Weathering processes in the Ganges–Brahmaputra basin and the riverine alkalinity budget. *Chem. Geol.* **159**, 31-60 (1999).

5. Molnar, P. et al. Geologic evolution of northern Tibet: results of an expedition to Ulugh Muztagh. *Science* **235**, 299-305 (1987).

6. Burg, J. P., et al. The Namche Barwa syntaxis: evidence for exhumation related to compressional crustal folding. *J. Asian Earth Sci.* **16**, 239-252 (1998).

7. Yang, K., et al. Recent climate changes over the Tibetan Plateau and their impacts on energy and water cycle: A review. *Glob. Planet. Change* **112**, 79-91 (2014).

8. Xu, Z., Gong, T. & Li, J. Decadal trend of climate in the Tibetan Plateau-regional temperature and precipitation. *Hydrol. Process.* **22**, 3056-3065 (2008).

9. Zhao, T. et al. The influence of carbonate precipitation on riverine magnesium isotope signals: New constrains from Jinsha River Basin, Southeast Tibetan Plateau. *Geochim. Cosmochim. Acta* **248**, 172-184 (2019).

10. Zhang, X. et al. Hydro-geochemical and Sr isotope characteristics of the Yalong River basin, eastern Tibetan plateau: Implications for chemical weathering and controlling factors. *Geochem. Geophys. Geosyst.* **20**, 1221-1239 (2019).

11. Négrel, P., Allègre, C. J., Dupré, B. & Lewin, E. Erosion sources determined by inversion of major and trace element ratios and strontium isotopic ratios in river water: The Congo Basin case. *Earth Planet. Sci. Lett.* **120**, 59-76 (1993).

12. Xu, Z. F. & Liu, C. Q. Chemical weathering in the upper reaches of Xijiang River draining the Yunnan -Guizhou Plateau, Southwest China. *Chem Geol* **239**, 83-95 (2007).

13. Gaillardet, J., Dupré, B., Louvat, P. & Allègre, C. J. Chemical and physical denudation in the Amazon

River basin. *Chem. Geol.* **142**, 141-173 (1997).

14. Viers, J. et al. Evidence for non-conservative behaviour of chlorine in humid tropical environments. *Aquat. Geochem.* **7**, 127-154 (2001).
15. Li., S. et al. Chemical balance of the Yellow River source region, the northeastern Qinghai-Tibetan Plateau: insights about critical zone reactivity. *Appl. Geochem.* **90**, 1-12 (2018).
16. Tang, J. et al. The preliminary study on chemical characteristics of precipitation at Mt. Waliguan. Acta *Sci. Circumst.* **20**, 420-425 (2000).
17. Jiang, H. et al. Chemical weathering of small catchments on the Southeastern Tibetan Plateau I: Water sources, solute sources and weathering rates. *Chem. Geol.*, **500**, 159-174 (2018).
18. Wang, X., He, K. & Dong, Z. Effects of climate change and human activities on runoff in the Beichuan River Basin in the northeastern Tibetan Plateau, China. *Catena* **176**, 81-93 (2019).
19. Li, Z. X. et al. Analysis on chemical compositions of rainwater in summer, Lijiang City, China. *Environ. Sci.* **30**, 362-367 (2019).
20. Zhang, N. et al. Long-term trends in chemical composition of precipitation at Lijiang, southeast Tibetan Plateau, southwestern China. *Atmos. Res.* **106**, 50-60 (2012).
21. Lin, W., Liao, D., Liu, Z. & Fan, H. Analysis on the change of acid rain and chemical characteristics of precipitation in panzhihua city. *J. Panzhihua Univ.* **29**, 120-123 (2012).
22. Li, C, Kang, S., Zhang, Q. & Kaspari, S. Major ionic composition of precipitation in the Nam Co region, Central Tibetan Plateau. *Atmos. Res.* **85**, 351-360 (2007).
23. Liu, Y. W. et al. Wet deposition of atmospheric inorganic nitrogen at five remote sites in the Tibetan Plateau. *Atmos. Chem. Phys.* **15**, 11683-11700 (2015).
24. Liu, B. et al. Wet precipitation chemistry at a high-altitude site (3326 m.a.s.l.) in the southeastern Tibetan Plateau. Environ. *Sci. Pollut. Res. Int.* **20**, 5013-5027 (2013).
25. Wang, W. et al. Chemical compositions of fog and precipitation at Sejila Mountain in the southeast Tibetan Plateau, China. *Environ. Pollut.* **253**, 560-568 (2019).
26. Noh, H., Huh, Y., Qin, J. & Ellis, A. Chemical weathering in the Three Rivers region of Eastern Tibet. *Geochim. Cosmochim. Acta* **73**, 1857-1877 (2009).
27. Zhang, D. et al. Sulfur cycling in the Yellow River and the sulfate flux to the ocean. *Chem. Geol.* **534**, 119451 (2020).
28. Relph, K. E. et al. Partitioning riverine sulfate sources using oxygen and sulfur isotopes: Implications

for carbon budgets of large rivers. *Earth Planet. Sci. Lett.* **567**, 116957 (2021).

29. Zhang, F. et al. Pollution characteristics and prospective risk of microplastics in the Zhengzhou section of Yellow River, China. *Sci. Total Environ.* **931**, 172717 (2024).

30. BryantMason, A., Xu, Y. J. & Altabet, M. Isotopic signature of nitrate in river waters of the lower Mississippi and its distributary, the Atchafalaya. *Hydrol. Process.* **27**, 2840-2850 (2013).

31. Johannsen, A., Dähnke, K. & Emeis, K. Isotopic composition of nitrate in five German rivers discharging into the North Sea. *Org. Geochem.* **39**, 1678-1689 (2008).

32. Hren, M. T. et al. Major ion chemistry of the Yarlung Tsangpo–Brahmaputra River: Chemical weathering, erosion, and CO_2 consumption in the southern Tibetan plateau and eastern syntaxis of the Himalaya. *Geochim. Cosmochim. Acta* **71**, 2907-2935 (2007).

33. Wu, W. H., Xu, S. J., Yang, J. D. & Yin, H. W. Silicate weathering and CO_2 consumption deduced from the seven Chinese rivers originating in the Qinghai-Tibet Plateau. *Chem. Geol.* **249**, 307-320 (2008).

34. Wu, W. H. Hydrochemistry of inland rivers in the north Tibetan Plateau: Constraints and weathering rate estimation. *Sci. Total Environ.* **541**, 468-482 (2016).

35. Gaillardet, J., Dupré, B., Louvat, P. & Allègre, C. J. Global silicate weathering and CO_2 consumption rates deduced from the chemistry of large rivers. *Chem. Geol.* **159**, 3-30 (1999).

36. Spence, J. & Telmer, K. The role of sulfur in chemical weathering and atmospheric CO_2 fluxes: evidence from major ions, $\delta^{13}C_{DIC}$, and $\delta^{34}S_{SO4}$ in rivers of the Canadian Cordillera. *Geochim. Cosmochim. Acta* **69**, 5441-5458 (2005).

37. Karim, A. & Veizer, J. Weathering processes in the Indus River Basin: implications from riverine carbon, sulfur, oxygen, and strontium isotopes. *Chem. Geol.* **170**, 153-177 (2000).

38. Singh, S. K., Sarin, M. M.& France-Lanord, C. Chemical erosion in the eastern Himalaya: Major ion composition of the Brahmaputra and $\delta^{13}C$ of dissolved inorganic carbon. *Geochim. Cosmochim. Acta* **69**, 3573-3588 (2005).

39. Li, X.Q., Liu, Y. D., Zhou, A. G. & Zhang, B. Sulfur and oxygen isotope compositions of dissolved sulfate in the Yangtze River during high water period and its sulfate source tracing. *Earth Sci.* **39**, 1547-1554 (2014).

40. Lerman, A. & Wu, L. CO_2 and sulfuric acid controls of weathering and river water composition. *J. Geochem. Explor.* **88**, 427-430 (2006).

41. Torres, M. A. et al. The acid and alkalinity budgets of weathering in the Andes-Amazon system: Insights into the erosional control of global biogeochemical cycles. *Earth Planet Sc. Lett.* **450**, 381-391 (2016).

42. Krouse, H. R. & Mayer, B. Sulphur and Oxygen Isotopes in Sulphate in *Environmental Tracers in Subsurface Hydrology* (eds Cook, P. G. & Herczeg, A. L.) 195-231 (Springer, 2000)

43. Liu, X. Y. et al. Assessment of atmospheric sulfur with the epilithic moss Haplocladium microphyllum: evidences from tissue sulfur and $\delta^{34}S$ analysis. *Environ. Pollut.* **157**, 2066-2071 (2009).

44. Calmels, D., Gaillardet, J., Brenot, A. & France-Lanord, C. Sustained sulfide oxidation by physical erosion processes in the Mackenzie River basin: Climatic perspectives. *Geology* **35**, 1003-1006 (2007).

45. Rock, L. & Mayer, B. Identifying the influence of geology, land use, and anthropogenic activities on riverine sulfate on a watershed scale by combining hydrometric, chemical, and isotopic approaches. *Chem. Geol.* **262**, 121-130 (2009).

46. Xu, Y., Liu, W., Xu, B. & Xu, Z., 2024. Riverine sulfate sources and behaviors in arid environment, Northwest China: Constraints from sulfur and oxygen isotopes. *J. Environ. Sci.* **137**, 716-731 (2024).

47. Yuan, F. & Mayer B. Chemical and isotopic evaluation of sulfur sources and cycling in the Pecos River, New Mexico, USA. *Chem. Geol.* **291**, 13-22 (2012).

48. Otero, N., Soler, A. & Canals, À. Controls of $\delta^{34}S$ and $\delta^{18}O$ in dissolved sulphate: Learning from a detailed survey in the Llobregat River (Spain). *Appl. Geochem.* **23**, 1166-1185 (2008).

49. Turchyn, A. V. et al. Isotope evidence for secondary sulfide precipitation along the Marsyandi River, Nepal, Himalayas. *Earth Planet Sc. Lett.* **374**, 36-46 (2013).

50. Feng, J. L., Chen, F. & Hu, H. P. Isotopic study of the source and cycle of sulfur in the Yamdrok Tso basin, Southern Tibet, China. *Appl. Geochem.* **85**, 61-72 (2017).

51. Burke, A. et al. Sulfur isotopes in rivers: Insights into global weathering budgets, pyrite oxidation, and the modern sulfur cycle. *Earth Planet. Sc. Lett.* **496**, 168-177 (2018).

52. Killingsworth, B.A., Bao, H., Kohl, I.E. Assessing Pyrite-Derived Sulfate in the Mississippi River with Four Years of Sulfur and Triple-Oxygen Isotope Data. *Environ. Sci. Technol.* **52**(11), 6126-6136.

53. Mukai, H. et al. Regional characteristics of sulfur and lead isotope ratios in the atmosphere at several

Chinese urban sites. *Environ. Sci. Technol.* **35**, 1064-1071 (2001).

54. Li, X. et al. Degradation of groundwater quality due to anthropogenic sulfur and nitrogen contamination in the Sichuan Basin, China. *Geochem. J.* **40**, 309-332 (2006).

55. Claypool, G. E. et al. The age curves of sulfur and oxygen isotopes in marine sulfate and their mutual interpretation. *Chem. Geol.* **28**, 199-260 (1980).

56. Turchyn, A. V.& Schrag, D. P. Cenozoic evolution of the sulfur cycle: insight from oxygen isotopes in marine sulfate. *Earth Planet. Sci. Lett.* **241**, 763-779 (2006).

57. Thode, H. Sulphur isotopes in nature and the environment: an overview, in *Stable Isotopes in the Assessment of Natural and Anthropogenic Sulphur in the Environment* (Eds Krouse, H. R. & Grinenko V. A.) 1-26 (John Wiley & Sons, 1991).

58. Strauss, H. The isotopic composition of sedimentary sulfur through time. *Palaeogeogr. Palaeocl.* **132**, 97-118 (1997).

59. Tian, S. H., et al. Sulfur, lead, strontium and neodymium isotope compositions of the Dongmozhazhua lead-zinc ore deposit in the Yushu area, southern Qinghai: implications for the sources of ore-forming material in the deposit. *Acta Petrol. Sin.* **27**, 2173-2183 (2011).

60. Wang, B. et al. Distinct patterns of chemical weathering in the drainage basins of the Huanghe and Xijiang River, China: evidence from chemical and Sr-isotopic compositions. *J. Asian Earth Sci.* **59**, 219-230 (2012).

61. Ding, T. P. et al. Chemical and isotopic characters of the water and suspended particulate materials in the Yellow River and their geological and environmental implications. *Acta Geol. Sin* **90**, 285-351 (2016).

62. Zhang, F. et al. The dominance of loess weathering on water and sediment chemistry within the Daihai Lake catchment, northeastern Chinese Loess Plateau. *Appl. Geochem.* **35**, 51-63 (2013).

63. Wang Z. L., Zhang J. & Liu C. Q. Strontium isotopic compositions of dissolved and suspended loads from the main channel of the Yangtze River. *Chemosphere* **69(7)**, 1081-1088 (2007).

64. Chetelat, B. et al. Geochemistry of the dissolved load of the Changjiang Basin rivers: anthropogenic impacts and chemical weathering. *Geochem. Cosmochim. Acta* **72**, 4254-4277 (2008).

65. Moon, S., Huh, Y., Qin, J. & Nguyen, V. P. Chemical weathering in the Hong (Red) River basin: rates of silicate weathering and their controlling factors. *Geochim. Cosmochim. Acta* **71**, 1411-

1430 (2007).

66. Chapman, H., Bickle, M., Thaw, S. & Thiam, H. Chemical fluxes from time series sampling of the Irrawaddy and Salween Rivers, Myanmar. *Chem. Geol.* **401**, 15-27 (2015).

67. Singh, S. K., Kumar, A.& France-Lanord, C. Sr and $^{87}Sr/^{86}Sr$ in waters and sediments of the Brahmaputra river system: Silicate weathering, CO_2 consumption and Sr flux. *Chem. Geol.* **234**, 308-320 (2006).

68. Bickle, M. J. et al. Relative contributions of silicate and carbonate rocks to riverine Sr fluxes in the headwaters of the Ganges. *Geochim. Cosmochim. Acta* **69**, 2221-2240 (2005).

69. Chakrapani, G.J. Major and trace element geochemistry in upper Ganga river in the Himalayas, India. *Environ. Geol.* **48**, 189-201 (2005).

70. Pande, K. et al. The Indus river system (India-Pakistan): Major-ion chemistry, uranium and strontium isotopes. *Chem. Geol.* **116**, 245-259 (1994).

71. Liu, W. J. et al. Chemical and strontium isotopic characteristics of the rivers around the Badain Jaran Desert, northwest China: implication of river solute origin and chemical weathering. *Environ. Earth Sci.* **75**, 1119-1135 (2016).

72. Zhang, J. et al. Water geochemistry of the rivers around the Taklimakan Desert (NW China): crustal weathering and evaporation process in arid land. *Chem. Geol.* **119**, 225-237 (1995).

73. Palmer, M. R. & Edmond, J. M. The strontium isotope budget of the modern ocean. *Earth Planet. Sci. Lett.* **92**, 11-26 (1992).
